# Teachers’ perception of their students’ dietary habits in Addis Ababa, Ethiopia: a qualitative study

**DOI:** 10.1186/s40795-024-00946-7

**Published:** 2024-10-22

**Authors:** Mekdes Mekonnen Kifle, Laura Terragni, Marianne Morseth

**Affiliations:** Department of Public Health Nutrition, Oslo Metropolitan University, St. Olavs plass, Oslo, 0130 Norway

**Keywords:** Dietary habit, Teachers’ perception, Healthy eating, Ethiopian students, Nutrition transition

## Abstract

**Background:**

Poor dietary choices and consumption of unhealthy foods are major determinants of malnutrition among adolescents in Ethiopia. The school food environment is a valuable setting for exploring adolescents’ eating habits. Teachers have an important role in understanding factors that impact students’ dietary choices. The aim of this study is to explore secondary school teachers’ perceptions towards adolescents’ dietary habits in Ethiopia.

**Methods:**

The study employed a qualitative research design. Four focus group discussions, involving a total of 13 teachers, were conducted at governmental and private schools in Addis Ababa, Ethiopia. Additionally, observations of the food environment in these four schools. The transcripts from the focus group discussions and photographs from observations were analyzed using thematic analysis. Triangulation of data sources and persistent observation of the data were employed to enhance the study’s trustworthiness. The study was approved by the Norwegian Center for Research Data and the Addis Ababa Health Bureau, and all participants provided informed consent.

**Result:**

Teachers perceived adolescents’ dietary habits as unhealthy, characterized by the consumption of unsafe foods, limited variety, and reliance on processed foods. Factors negatively influencing adolescents’ dietary habits include a lack of awareness about a healthy diet among both students and parents. Low familial income levels were also identified as a barrier to eating a healthy diet. The unavailability of healthy foods and the advertising of unhealthy and processed foods as well as peer influence were hindrances to a healthy diet both at school and home.

**Conclusion:**

This study provides additional evidence of the nutrition transition which is linked to the double burden of malnutrition among adolescents in low-income countries. Factors affecting adolescent diets at school are multileveled. Incorporating nutrition education into the school curriculum will likely improve dietary awareness mitigating peer influence. Regulating the school food environment and enforcing advertisement laws targeting adolescents can promote healthier school food environments. Providing short term nutrition trainings for biology or science teachers and strengthen their role in delivering nutrition education to children and their families, along with implementing measures to address food insecurity and restricting availability of unhealthy food at school need to be regarded as priorities.

**Supplementary Information:**

The online version contains supplementary material available at 10.1186/s40795-024-00946-7.

## Background

In the past decades, there has been a significant and dramatic shift in dietary patterns worldwide [1]. Globalization and trade policies are deeply transforming societies, shaping political institutions, economic and social relationships, modes of food production, food consumption patterns and lifestyles [2]. These structural factors have been identified as potentially important drivers of rapid dietary changes [3], and linked to malnutrition in all its forms [1]. Starting in high income countries, a westernized diet pattern has emerged with traditional, largely plant-based diets being replaced by diets high in animal products, fat, highly processed foods, added sugar and salt. This dietary shift, known as the nutrition transition, is accompanied by a shift towards more sedentary work and leisure patterns [4].

Unhealthy diets predispose people to malnutrition and diet-related non communicable diseases (NCDs) such as obesity, type 2 diabetes and cardiovascular disease, which increase the risk of early mortality [5–7]. Nutrition related conditions are increasing globally, while high income countries are characterized by an increase in diet-related NCDs [5, 6], low- and middle- income countries (LMICs) are increasingly facing a triple burden of malnutrition [8, 9].

A food environment is the physical, economic, political, and sociocultural context which affects people’s food choices [10]. School food environments are valuable settings for exploring the nutrition transition, and for influencing healthy eating habits among adolescents [11, 12]. Children and adolescents spend more time in schools than in any other environment away from home, indicating the potential influence of the school environment on dietary habits [11, 13, 14]. This makes the school a convenient setting to teach about heathy eating habits. Schools may also play an important role in creating a healthy food environment that provides students with nutritious and appealing foods and beverages [12]. Consistent and accurate messages about good nutrition and healthy eating practices can also be provided in the schools [15–17].

For sustainable changes and improvement in the food environment, interventions should target children from an early age, involving family/parents, teachers and peers as role models [18]. Teachers are uniquely positioned to provide insight into their students’ dietary habits [13, 19]. Teachers can also exert a significant influence on students’ eating habits and health-related behaviors given their close proximity and interaction throughout the school day [20].

Studies indicate that unhealthy eating habits such as skipping meals, consumption of highly processed foods and sugar-sweetened beverages, and poor fruit and vegetable intake are common among adolescents [21–23]. Several factors impact adolescents’ dietary habits and food choices, including family, school and media [24]. In a qualitative study conducted in Kolkata, India in 2020, parents and educators suggested that the main reasons for adolescents’ unhealthy food consumption are proliferation of fast food retail outlets, hyper-palatability of fast food, marketing of fast food in electronic and social media, diminished household cooking practices, lack of food knowledge, and tendency to seek peer group acceptance [25].

In Addis Ababa, there are over 140,000 secondary school students, of which 54.1% are female [26]. As of 2016, there were 2,154 schools, including 462 government-funded, 28 public, and 1,664 privately owned schools [27]. Secondary students typically spend seven hours at school and have two meal breaks. Students either bring food from home or buy it from nearby food vendors. A program entitled ‘School Feeding Program (SFP)’ was initiated in Addis Ababa in February 2019, aiming to provide meals to children in primary schools [27]. Teachers can play a significant role in influencing their students by including lessons on nutrition modeling healthy eating behaviors [20].

Ethiopia’s food systems are rapidly evolving. Significant diet changes include higher total calorie consumption, a declining share of starchy staples and an increasing share of fruits and vegetables [28]. Frequent consumption of processed convenience and out-of-home foods is becoming more common. Poor dietary choice and consumption of unhealthy foods is found to be one of the major determinants of malnutrition among adolescents in different regions of Ethiopia [29–32]. Considering the complex dietary behaviors and the diversity of influences on diet, it is essential to assess the factors which impact adolescents’ dietary choices. This study aimed to investigate teachers’ perceptions of adolescents’ dietary habits in Ethiopia. A socioecological model was used to describe the factors associated with food choices among adolescents. This model emphasizes the interaction between factors across all levels of health behavior [33]. Furthermore, the findings from this study can contribute to the development of robust interventions which promote healthy dietary practices among adolescents.

## Methods

### Participants and setting

This study was conducted in four schools in Addis Ababa, Ethiopia. Two sub-cities were chosen from a total of 11 sub-cities in Addis Ababa, one representing areas of higher socioeconomic status and the other representing relatively low socioeconomic status. In each selected sub city, one private school and one governmental school were chosen based on the recommendation of the sub-city education bureau. These schools have been selected to obtain a heterogeneous sample of teachers from private and public schools, thus enriching the findings of the study. Government schools are fully funded and administered by government [34]. These schools offer education for free, while private schools charge a fee for their services but often have superior facilities and more highly educated teachers [35].

A purposeful sampling technique was employed [36], with the assistance of school unit leaders, to identify teachers eligible to participate in the study. Purposeful sampling was used to recruit information-rich samples which were selected based on relevant characteristics related to the topic of discussion [37]. All participants were teachers of different subjects (language, biology, geography) for grade 11 and 12 students, which makes them representative. Choosing teachers of different subjects also helps avoid biased perceptions due to prior knowledge, which could occur if all the teachers were from the same department, such as biology, which is more related to nutrition. These grades were chosen because students at this level begin to make more independent food choices and are generally more autonomous compared to younger students [38].

Food vendors and shops in the vicinity of each school were included in the study for observation. Furthermore, food vendors identified by the teachers as popular among students were also observed to complement their point of view. Most food vendors sell unhealthy foods such as donuts, French fries, biscuits and soft drinks.

### Design and data collection

In this qualitative study, focus group interviews were utilized to explore teachers’ perceptions regarding students’ food choices and the factors influencing their dietary decisions. For each school, one focus group was conducted, comprising 3–4 participants and two facilitators. The facilitators included the first author, a female medical doctor with previous research experience and another trained assistant. All focus group discussions were conducted face-to-face within the school compound and field notes were taken during the interview. The number of focus groups and sample size were decided based on the concept of code saturation [37, 39].

The focus group interview guide (Appendix 1) was developed by the authors and piloted before beginning data collection. Minor adjustments were made after the pilot focus group interview and pilot interviews were not included in the analysis. During the interviews, facilitators played the role of listeners, allowing the teachers to freely express their perspectives without influencing them to agree or disagree with others’ ideas. This study also employed observation to investigate the school food environment. The food environment of each school, including the foods that are available and mostly consumed by the students when they leave the school grounds was observed and photographed by the first author of the study over three days. Data collection took place from November 2022 to January 2023, and the duration of each observation and focus group session lasted 30 min on average.

### Data analysis

All focus group discussions were digitally recorded and transcribed verbatim to retain details and ensure reliability. After transcribing the data, the full text was translated from Amharic to English language, first using google translate and then revised for accuracy by the primary investigator who is fluent in both languages. A codebook was developed based on the existing literature, structuring the factors in to the four out of the five domains (intrapersonal, interpersonal, organizational and community level) of the socioecological model [33]. The coding was conducted using NVivo 12 [40]. The preliminary codes were discussed among co-authors to finetune results and identify alternative interpretations. The first Codes that exhibited interconnectedness and cohesiveness were identified through visual representations supplemented by an overarching theme. Subsequently, these distinct codes were categorized into prospective themes, with all pertinent coded data extracts aggregated within these identified themes. Initially, four themes were identified: “The current dietary practices,” “Changes in dietary habits,” “Factors influencing healthy dietary practices,” and “Strategies to improve dietary habits.” The emerging themes were mostly similar for both governmental and private schools. Following the formulation of themes, the codes corresponding to pertinent text segments from interviews, aligning with each theme, were systematically identified, and arranged to achieve the most meaningful organization. Thematic analysis as described by Braun and Clarke was employed as the method of analysis incorporating both inductive and deductive approaches [41]. The deductive approach utilized a theory driven framework (in this case, the socioecological model) for identification and classification of relevant themes. In addition, inductive coding was used for capturing subthemes emerging from the data. The observation results are analyzed according to the type of school and presented as food environment in government and private schools.

Before initiating the study, ethical approval was granted by the Norwegian Center for Research Data (Reference No: 140722) and the Addis Ababa Health Bureau (Reference No: A/A/3836/227). Prior to the commencement of data collection, the purpose of the research was explained both orally and in writing, and informed consent was received from all participants.

### Trustworthiness of the study

In this research, efforts were made to establish credibility through persistent fieldwork, ensuring alignment between the findings and reality. Additionally, the researcher accurately recorded the focus group discussions to obtain a detailed description of the situation. Another method used to ensure credibility was the persistent observation of the data, where the researcher read and reread the data during transcription, translation, and coding. Methodological triangulation (focus group discussions and observations) was employed to enhance the validity of this study.

To ensure the transferability of the findings, rich and thick descriptions of the focus group discussions among teachers were provided, including details on where the research was conducted, its setting, sample, sample size, sampling strategy, demographics, interview procedures, and topics. To enhance the dependability and confirmability of this study, a comprehensive and rational portrayal of the study’s perspective, the researcher’s involvement, the informants’ stance, the location where data was gathered, the research design, data collection methods, and data analysis techniques has been provided.

## Results

A total of thirteen teachers participated in this study, seven from governmental schools and the rest from private schools. All participants were teachers of grades 11 and 12, and all but one were male.

### Teachers’ perception regarding their student’s dietary practice

While at school, teachers noticed that most of the students consumed different types of sweet foods like donuts and cakes as snacks, as well as pasta, ‘ertb’ (a sandwich made with potatoes), ‘injera’(a flat bread made from ‘teff’, teff is a cereal cultivated in Ethiopia and Eritrea. The batter for injera undergoes a fermentation process for several days then poured into a large round griddle called “mitad” and cooked until bubbles form on the surface. ) with stew, and ‘fır-fır’(Ethiopian food prepared from shredded ‘injera’ for lunch. A few teachers mentioned that students also consume fried foods like French fries and prepackaged foods while others noted students eating eggs, potato stew, bread and ‘kita’ (a special thin bread made from wheat). Sugar-sweetened beverages, tea, water, and coffee were the most consumed drinks within the school. At one private school, students were banned from drinking sugar-sweetened beverages on the school premises. The teacher described it as follows:

*“In our school*,* each student brings his own food*,* mostly ‘injera’ made from ‘teff’; ‘fir-fir’; spaghetti*,* penne. Also*,* when we say balanced food*,* it should contain vegetables and fruits*,* every day from Monday to Friday*,* they bring these three foods that I mentioned earlier. Then use bottled water*,* they buy bottled water from school… There is even purified water… They drink that water… Any other drink such as Coca-Cola; Miranda; Pepsi; Ambo mineral water is not allowed in the school compound*.” (PS, FG10).

**Fig. 1:**
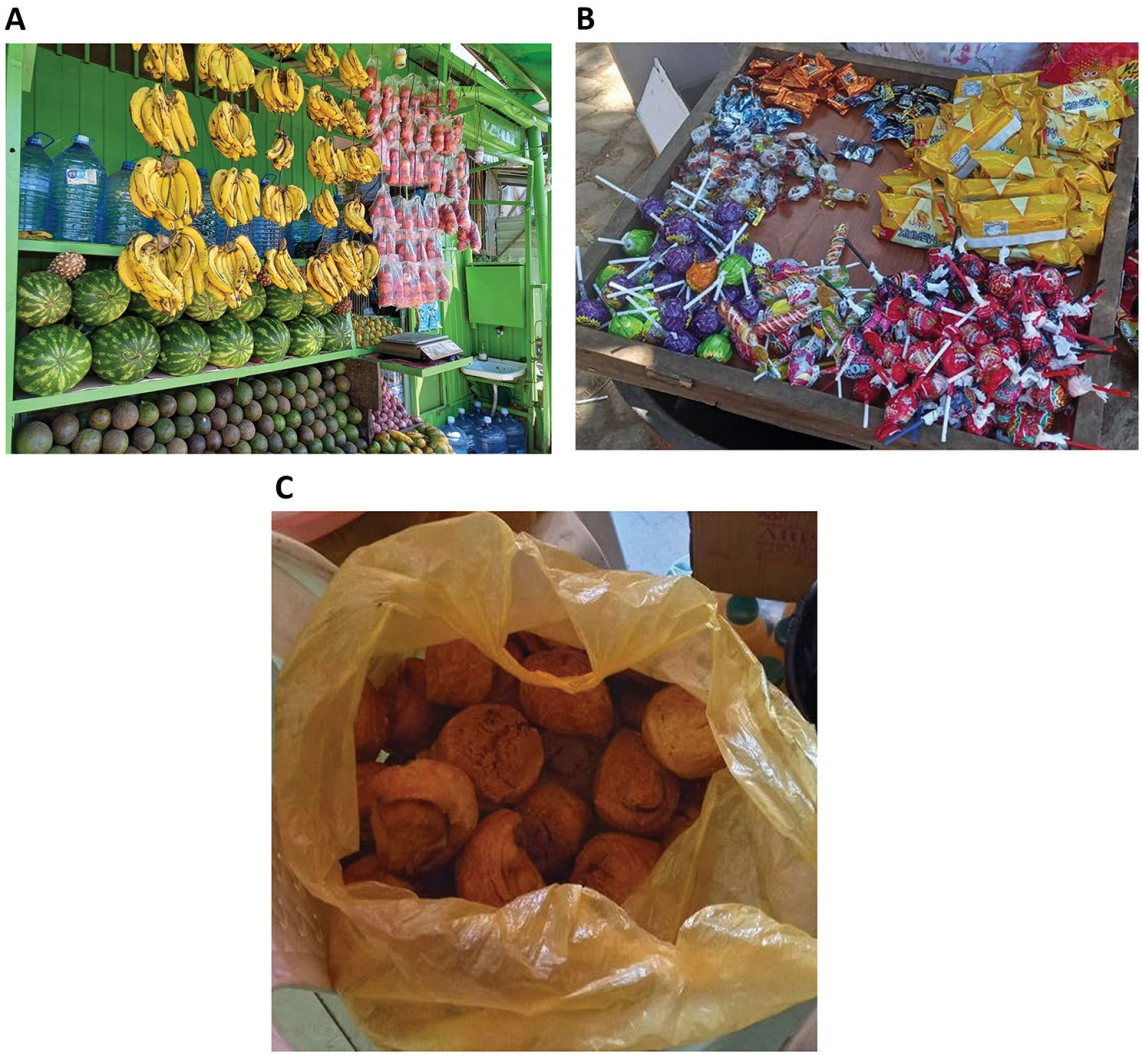
Food environment of government schools. **A**. (Upper picture) fruits, **B**. (middle picture) candies, lollipops and biscuits, **C**. (Lower Picture) ’koker’

**Fig. 2:**
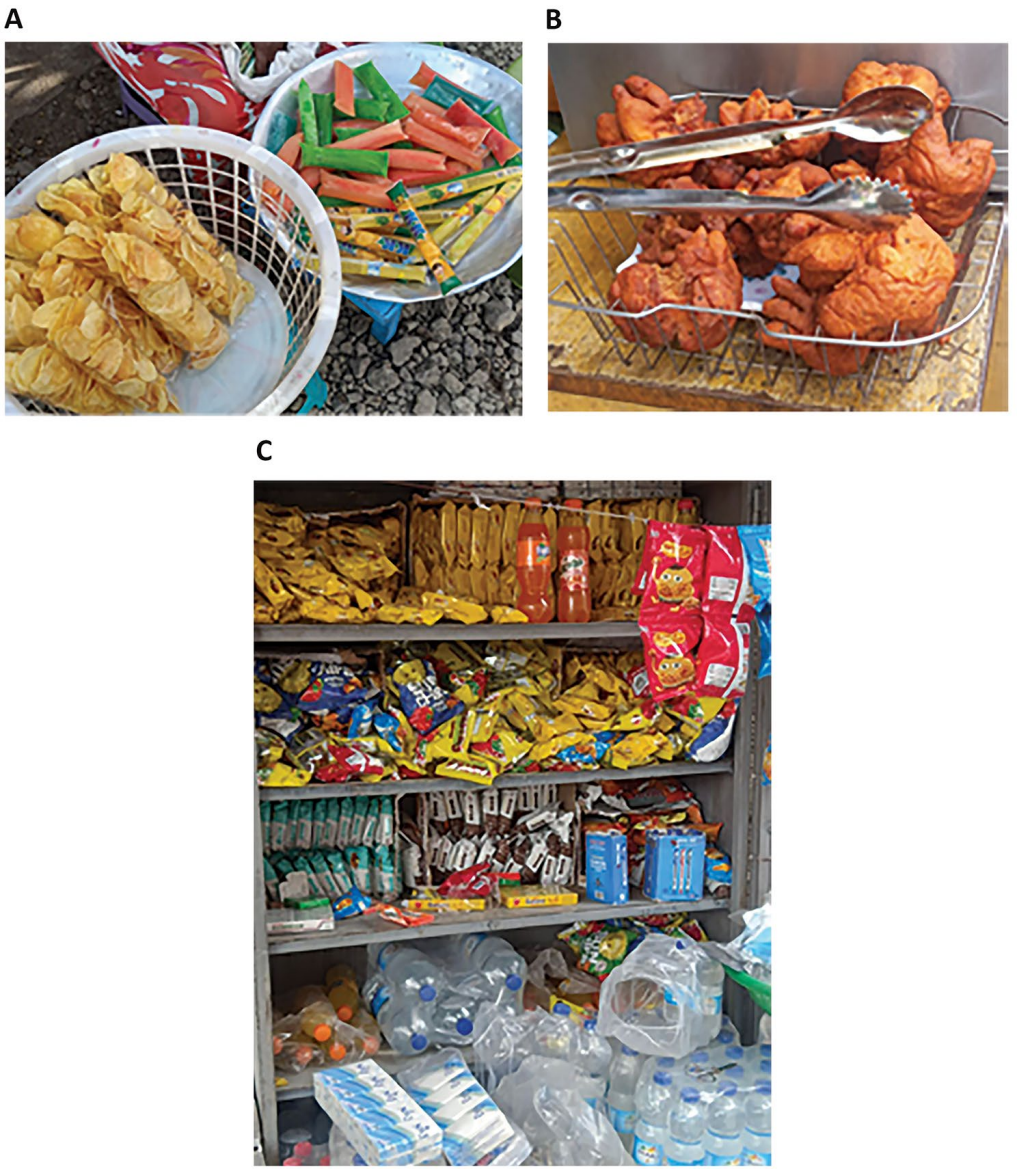
Food environment of private schools. **A**. (Upper picture) Chips, Candy **B**. (middle picture) ’Koker’ **C**. (Lower Picture) Biscuits, chips, sugar-sweetened beverages

Observation of the school food environment revealed that fruits and vegetables were available within 3–4 min walking distance in only one of the four schools. Fruits and vegetables were only sold by weight, not as individual pieces. In the remaining schools, fast foods, sugar-sweetened beverages, and sweets were readily available and positioned prominently to catch the attention of the students. Figures 1 and 2 show the school food environment in the governmental schools and private schools respectively.

Teachers did not only observe what students eat but they also made judgments and expressed concerns about the lack and scarcity of food. In one governmental school, a teacher was alarmed by students who had nothing to eat. One teacher described it as:

*“ In fact*,* I think some of them don’t bring food*,* there are occasions when they go around and share food from their friends. There are some who come to school without eating any food*,* so they simply drink water in the school not to fall (from lack of energy) or not to have bad mouth odor. I observed that there are children who spend their days drinking only water.”* (GS, FG9).

Although some teachers regarded the students’ diet as healthy, especially when it involved home-cooked meals, several teachers from various schools perceived the students’ dietary habits as unhealthy, citing limited variety, and reliance on processed foods. One teacher from a government school provided the following description :

“…*It is hard to say they are eating healthy food*,* If they are eating balanced food*,* what they eat should have variety. It’s hard to take what they are eating here*,* a balanced diet. As they eat the same food for a whole week*,* ”* (GS, FG12).

In addition, one teacher raised concerns regarding the consumption of improperly handled or stored foods:

“……*food that is prepared in school or bought and used in a café*,* is touched with hands repeatedly*,* it is exposed to contaminants as it is not covered*,* so the food may not be healthy*.” (GS, FG12).

One teacher mentioned the difference in dietary practices based on socioeconomic status as follows:

“*From the point of view of our school*,* you can divide their eating habits in three ways. There are some who come from families with good socioeconomic status and are properly cared for. They bring food which has variety*,* and which is prepared appropriately. There are some who don’t bring food*,* but they bring money so that they can buy food in the school… they often buy candy or biscuits and eat that*,* and then there are those who don’t have anything else to eat.*,* Most of the children who come to us are those who are not able to afford it…”* (GS, FG9).

### Barriers for practicing a healthy diet

#### Intrapersonal factors

Teachers from both governmental and private schools perceived that students’ lack of awareness and understanding about the healthiness of food may be an obstacle to eating a healthier diet. Teachers specifically mentioned that students preferred food bought at school rather than eating a healthier alternative brought from home as illustrated in the following quote :

“*I think the obstacles are probably a matter of understanding. The children themselves have their own desire to eat the food which is prepared here (in the school) instead of the food they bring from home… I think there is a lack of awareness. It is not due to lack of options.”* (PS, FG10).

In addition, another teacher from a governmental school highlighted concerns regarding the spending habits of students, particularly regarding their food choices purchased. This was also related to poor knowledge and understanding of healthy dietary practices among students as illustrated in the following quote:

*“ The students themselves… They earn money… But they don’t spend the money they earn properly*,* they don’t plan how to spend*,* for example*,* what kind of food they should eat if they have to buy food*,* even based on their age*,* they often consume ‘bombolino’(snacks made from wheat flour) and sugar-sweetened beverages as it is mentioned above*,* they don’t know about health and healthy diet… They don’t plan where to spend that coin properly; they are exposed to these kinds of problems and various diseases.”* (GS, FG3).

#### Interpersonal factors

Parental barriers to eating a healthy diet were also identified and included insufficient parental care as well as inadequate parental awareness and understanding of healthy dietary practices. One teacher mentioned:

“… *but I think that the students who study here are mostly under 18 years old*,* so their parents are the ones who guide these children*,* I think the problem is with the parents. It is the parent who can choose the food that is good for their children…Parents should not only create awareness*,* but it is also better if parents prepare food*,* the parent can choose the food better than the student… So*,* I think it’s a lack of parental awareness.”* (PS, FG10).

Additionally, governmental schoolteachers mentioned that a limited family income level may contribute to poor dietary practices due to inadequate food quantities and a lack of dietary diversity, as reflected in the following quote:

*“One of the things that can prevent people from having a healthy diet*,* as it is being said*,* is the economy… let alone a balanced meal*,* there are those who do not bring food to eat. That is a problem. Again*,* even those who bring a balanced meal… it has limitations on how much they can eat and the amount to eat from the different types. The main thing that causes them not eat a balanced meal is having low family income.”* (GS, FG12).

Some students from economically disadvantaged families are forced to work to help support their families. One teacher from a government school described how this economic challenge can further create barriers to practicing a healthy diet, due to time constraints, and competing priorities:

*“ …there are those who support their families unfairly. They try to help their families by doing different jobs*,* for example carrying different things*,* selling secondhand clothing. This might create pressure when they come here (to school). It makes them not take care of themselves*,* and prevents them from eating a healthy diet…”* (GS, FG9).

Peer pressure and the preference to eat food away from home with friends were also mentioned as barriers to a healthy diet. A private school teacher explained it as:

“ *Now*,* for example*,* if the lunch and breakfast that the children have is different from that of another friend they leave without eating or they may throw it (their lunch) in the garbage can. If they start saying they don’t want to take (the food) when it is put in the lunch box at home*,* it might be due to the food that their friend brings.”* (PS, FG11).

#### Organizational factors

Teachers from different schools emphasized the easy access of unhealthy food options within the school food environment as possible obstacles to students’ healthy food decisions. The school food environment in private school was described by one teacher as:

*“There are shops open around the school and*,* there are things being sold*,* It is student-centered*,* not from a health perspective*,* but the merchant does it from an income perspective. So they sell a lot of sweet things near the school. I think that it has more impact. Even if students try hard at home*,* the things they find outside will have an impact on their health.”* (PS, FG11).

#### Community level factors

Teachers observed changes in the food preparation methods used by the community. They explained that previously ingredients such as, spices, were prepared at home, whereas now they are being bought from the market. One teacher reflected the changes towards more processed foods:

*“… diet has changed a lot now. Natural things are disappearing*,* while more and more things are processed in factories. When something goes in and out of a factory*,* there are different ingredients added… There is an effect caused by the added ingredient chemicals. They are unnatural. ”* (PS, FG11).

Other teachers mentioned food advertisements as possible obstacles for students to eat a healthy diet. Teachers described the frequent overstatements in food advertisements as a potential barrier. :

*“ The food advertisements which we see on social media*,* and other mainstream media that are advertised for their age group and which is a deviation from the regular diet. Most of them are sweet things which are advertised as if important and energy boosting even it is advertised by showing unrelated concepts…”* (PS, FG11).

### Suggested measures to improve student’s dietary habits

Most teachers mentioned increasing awareness as a possible solution to improving the students’ current dietary practices. Integrating nutrition lessons into the school curriculum was suggested as one way to increase knowledge about choosing and preparing a healthy diet based on income. The teachers emphasized that nutrition training sessions should not be limited to students alone but should also involve their families. Knowledgeable parents would be better able to influence their children’s eating habits. A private school teacher ongoing nutrition education within schools provided by government health officials:

*“Health experts in the Ministry of Health should raise awareness in schools at different times*,* not only providing (nutrition education) once and running away*,* but also (providing nutrition education) multiple times about nutritional status*,* the benefits of nutrition*,* how to prevent disease*,* how to fight disease….”* (PS, FG10).

Both governmental and private school teachers agreed on the importance of making the school food environment healthy for students. Urban agriculture and farming in school programs were also recommended for addressing the issue of availability of healthy food in the school. Extending the school meal program was another way of providing healthy meals mentioned by three governmental schoolteachers:

*“The second option is that our school is large. If there is a feeding program*,* one can plant vegetables in the school compound which can provide (healthy) food which might influence eating habits… It’s possible to buy cows in our school to provide milk for the students. It is also possible to breed chickens as we have a large yard in the school compound.”* (GS, FG 9).

Reducing the workload on the children outside of school and working hard to improve the economic situation of the family could help to alleviate the problem. Interestingly one teacher from a private school raised reconciliation of the current and past dietary habits across generations as an important intervention point:

*“ … now it is to reconcile the diet of this era and the diet of the past. Sometimes there are times when children guide. For example*,* I have valid knowledge about nutrition. But if I listen to my child*,* there is a child’s need and an adult’s knowledge*,* and work must be done to reconcile the two.”* (PS, FG11).

The development and implementation of dietary policies were also mentioned as important measures to improve current dietary habits :

*“A knowledge-based solution is needed. A general guide is needed. A food safety guide*,* an advertising guide is required. If you have seen it now*,* in the advertisement they eat a biscuit and do acrobatics. So if you say to your child*,* don’t eat (that biscuit) what he sees and hears will be different. So*,* advertisement law is needed. The law regulates the safety and quality of manufactured products*,* but it is not implemented*,* or the executive body does not do that.”* (PS, FG11).

Collaboration among all responsible stakeholders, including health professionals, non-governmental organizations, investors, and the agriculture sector was proposed as a solution to improving students’ diets. Private school teachers suggested the holistic change should involve students and the consumers at the individual level:

*“I don’t expect (change) from the top. Every individual*,* students*,* everyone should contribute to improve the students dietary habit …… Starting from the top*,* when the politics improves; when agriculture improves otherwise there is no place for nutrition to shine and be improved. because these are related things………”* (PS, FG11).

## Discussion

This study was conducted to explore teachers’ perceptions of their students’ dietary habits in Addis Ababa, Ethiopia. The findings of this research provide insights into factors influencing food choices among adolescents with possible solutions for improving the current dietary habits.

According to teachers, students frequently bring foods lacking vegetables and fruits, making it difficult to consider their diets as healthy. Consumption of unclean food was also mentioned as a contributing factor to the students’ unhealthy diets. Previous studies suggest that several factors contribute to unhealthy eating, including the unavailability, lack of access and unaffordability of certain foods [42–44]. These elements fall under the category of environmental factors, which influence adolescents’ dietary behavior.

Lack of knowledge is another factor which contributed to students’ unhealthy diets. According to the socioecological model, knowledge is an intrapersonal factor, which influences food choice. Studies conducted in various parts of the world suggest that nutrition knowledge and literacy is an important determinant for healthy dietary habits among adolescents [42, 45–48] which supports our study findings. Improving food and nutrition literacy of all Ethiopians is an objective of the national nutrition program [49]. Emphasizing nutrition education in the school curriculum allows teachers to instruct students about healthy eating, thereby increasing awareness and positively influencing adolescents’ dietary choice [50].

Adolescents’ dietary choices were significantly influenced by their peers, a discovery consistent with prior research [43, 51–54]. Raising awareness helps mitigate the impact of peer influence, enabling individuals to make well-informed decisions [55]. Furthermore, having teachers and parents as role models who engage in healthy eating behavior can motivate and encourage learners to adopt healthy eating behavior themselves [50]. Supportive teachers can also encourage adolescents to seek guidance and ask questions about their eating habits [56].

Low parental nutrition awareness was also considered a barrier to adolescents eating a healthy diet, and is supported by previous studies [57]. Raising awareness at the community level was one solution recommended by teachers for improving students’ eating habits. The United Nations Food and Agricultural Organization (FAO) recommends a holistic integrated, multi-sectoral and multi-disciplinary approach to end hunger and ensure food security [58]. The ministry of health, ministry of education, and ministry of agriculture should work together to improve the nutrition related burden among adolescents as it is crucial to halt the double burden of malnutrition [50]. The Ministry of Health and Education can plan and implement nutrition and health education provided at different grade levels. All three ministry offices could also collaborate on the implementation of a school food program to improve dietary practices among adolescents.

Advertisements were identified as a barrier to healthy eating habits among adolescents, as they encourage the purchase of advertised products [59]. Unfortunately, these advertisements often promote unhealthy and processed foods, targeting adolescents as independent consumers [52]. In the 21st century, when adolescents are exposed to numerous advertisements in their physical environment and on various social media platforms, promoting and enforcing advertising laws and regulations is crucial [60]. Banning advertisements and limiting the availability of sugar-sweetened beverages, which was seen in one private school, is also supported by previous studies [50, 59]. This commendable initiative could also be implemented by other schools to mitigate the influence of environmental factors which lead the students towards unhealthy food choices. A sugar tax to discourage the consumption of sugar-sweetened beverages has not yet been implemented in Ethiopia, but it might be an effective policy to decrease the purchase and consumption of SSBs [61].

Income is an important influence on students’ dietary habits [42]. The differences between private school and public-school students became apparent from the interviews. Food insecurity determinants like affordability, availability and accessibility are highly tied to the economic level of a family, and a community as well as a country [62]. A stronger national economy may lead to higher familial income which increases access and affordability of healthier food options such as fruits, vegetables, and legumes. It can also provide funding for public health campaigns that can raise nutrition awareness within the schools.

Private school teachers identified school food environments which promote excessive consumption of unhealthy foods as potential barriers to healthy eating. This study found that the school food environment was primarily characterized by fast food and sugar sweetened beverages. These findings are also supported by other studies of school food environments [42, 63]. Nutritious food options were also lacking. While fruits and vegetables were available in one of the four school food environments studies, they were only sold by weight, making them too expensive for most students who could only afford smaller portions. This indicates that teaching adolescents about healthy diet is not enough to change their dietary behavior. Rather, future intervention should address all the intrapersonal, interpersonal, organizational and community level factors to reach the desired outcome. For example, making fruits and vegetables accessible and affordable for students.

Interventions are urgently needed to improve the accessibility and affordability of healthy diets to abate the double burden of malnutrition among adolescents [64]. In this study, urban agriculture, farming in school, and school meal program were identified as possible solutions to improve the dietary habits of adolescents. Improving the school food environment by including more healthy foods options can have a positive impact on eating habits. It can help in reducing the consumption of unhealthy foods, further preventing the development of obesity and other diet-related diseases [42, 43].

Teachers perceived a shift in students’ food preferences towards processed, high-sugar, and fried foods, which contrasts with what students were eating a few years ago. The students’ current dietary habits are consistent with the beginning of the nutrition transition, incorporating many high energy and sugary foods [65, 66]. This eating pattern may be explained in part by the urban study setting. The ideas that urbanization is related to the nutrition transition, and that adolescents are highly engaged in unhealthy dietary behavior are also supported by studies conducted in different countries [67–70]. Students’ preference for processed foods can also be attributed to globalization, and to the influence of adolescents by celebrities through advertisements on social media to adopt western dietary habits [71]. This highlights the significant scope of the problem.

The influence of the nutrition transition was not limited to the students’ food choices, but also affects food preparation methods at home and at the food industry which makes it difficult to ignore its affect. While it is not feasible to prevent globalization, educating students about a healthy diet is feasible and can empower adolescents to navigate and adapt in an evolving food environment. Additionally, the development of national food policies and regulations can help mitigate the impact of globalization on dietary practices [72].

This study aimed to investigate the teacher’s perception of their students’ dietary habits using two different methods: focus group discussion and observation. The focus group discussions were conducted to describe the students’ dietary practice from the teachers’ point of view. The use of triangulation by combining forms of data collection can be considered a strength of this study. A separate study was conducted among adolescents in the same school, which also contributed to strengthening the research design.

During the analysis process, this study identified factors affecting dietary choices including parents. We would have provided a more comprehensive understanding if the study had explored parental perceptions and attitudes as well as the perspectives of the responsible governmental officials. This is considered as a limitation of this study as it would have helped to gain a holistic view of the subject. Seasonal variation in the availability and accessibility of certain fruit and vegetable items is also considered as another limitation of this study, given the two-month timeframe for data collection.

Sampling was biased towards male teachers, who were more committed to the study. Including only 13 participants and four focus group discussions in the study due to time constraints can also be considered a limitation. The study provides important insights to the factors influencing students’ dietary behaviors in LMICs, but the findings may not be transferred to rural areas in Ethiopia. It is recommended that further studies be conducted to obtain a more complete picture of the factors affecting healthy dietary practices, especially in rural settings.

## Conclusions

Teachers noticed the change of dietary habits towards more processed foods which is in line with the current global nutrition challenge that is the nutrition transition. The school food environment is more conducive for unhealthy dietary habits due to the availability and promotion of unhealthy food options such as French fries, biscuits, and ‘ertb’ and soft drinks. Conversely, there is a lack of fruit and vegetables near the school compound. Although these items are present in one school environment out of four schools, they are sold in bulk (Kilo) rather than individual pieces, making them inaccessible for the students.

Our study findings suggest that increasing awareness of healthy diets can be achieved by integrating nutrition courses into the school curriculum and initiating farming programs in schools and urban areas, thus promoting food security. School is an important arena for promoting awareness of adequate nutrition and healthy diets. In low-income countries like Ethiopia, schools can contribute to addressing the double burden of disease. Strengthening the role of teachers in providing nutrition education to children and their families, together with measures to address food insecurity, needs to be regarded as a priority. The health, education, and agriculture sectors, as the main responsible bodies for ensuring nutrition awareness and promoting healthy dietary practices among adolescents, should work diligently to implement the objectives of the national nutrition program.

Moreover, the development and implementation of dietary policies and improving the current economic situation can improve the dietary habits of adolescents. In general, a holistic change with the involvement of all stakeholders is required to correct unhealthy dietary practice.

## Electronic supplementary material

Below is the link to the electronic supplementary material.

Supplementary Material 1

## Data Availability

The data sets used and /or analyzed during the current study are available from the corresponding author on reasonable request.

## Abbreviations

FG: Focus group
GS: Government school
LMICs: Low- and middle-income countries
NCDs: Non communicable diseases
No: Number
PS: Private school
SFP: School feeding program

## References

[1] Wighton K, Imperial News. Global diets have seen dramatic changes over past 50 years, reveals study | Imperial News | Imperial College London. 2020. Accessed May 1, 2023. https://www.imperial.ac.uk/news/194713/global-diets-have-seen-dramatic-changes/

[2] Cuevas García-Dorado S, Cornselsen L, Smith R, Walls H. Economic globalization, nutrition and health: a review of quantitative evidence. Global Health. 2019;15:15. 10.1186/s12992-019-0456-z. 30786909 10.1186/s12992-019-0456-z PMC6381642

[3] Walls H, Baker P, Parkhurst J. Addressing trade policy as a macro-structural determinant of health: the role of institutions and ideas. Global Social Policy. 2018;18(1):94–101. 10.1177/1468018117748700.

[4] Popkin BM, Adair LS, Ng SW. NOW AND THEN: The Global Nutrition Transition: the pandemic of obesity in developing countries. Nutr Rev. 2012;70(1):3–21. 10.1111/j.1753-4887.2011.00456.x. 22221213 10.1111/j.1753-4887.2011.00456.x PMC3257829

[5] Rippe JM, editor. *Lifestyle Medicine*, Third Edition. 3rd edition. CRC Press; 2019.

[6] Roser M, Ritchie H, Spooner F. Burden of disease. *Our World in Data*. Published online September 25, 2021. Accessed May 16, 2023. https://ourworldindata.org/burden-of-disease

[7] Willett W, Rockström J, Loken B, et al. Food in the Anthropocene: the EAT–Lancet Commission on healthy diets from sustainable food systems. Lancet. 2019;393(10170):447–92. 10.1016/S0140-6736(18)31788-4. 30660336 10.1016/S0140-6736(18)31788-4

[8] World Health Organization [WHO]. The double burden of malnutrition: policy brief. 2017. Accessed May 29, 2023. https://www.who.int/publications-detail-redirect/WHO-NMH-NHD-17.3

[9] Prentice AM. The Triple Burden of Malnutrition in the era of globalization. Nestle Nutr Inst Workshop Ser. 2023;97:51–61. 10.1159/000529005. 37023735 10.1159/000529005

[10] Fanzo J, Davis C, Food Systems. Food environments, and Consumer Behavior. In: Fanzo J, Davis C, editors. Global Food Systems, diets, and Nutrition: linking Science, Economics, and policy. Palgrave Studies in Agricultural Economics and Food Policy. Springer International Publishing; 2021. pp. 9–28. 10.1007/978-3-030-72763-5_2.

[11] Wilf-Miron R, Kittany R, Saban M, Kagan I, Saban M. Teachers’ characteristics predict students’ guidance for healthy lifestyle: a cross-sectional study in arab-speaking schools. BMC Public Health. 2022;22:1420. 10.1186/s12889-022-13795-5. 35883162 10.1186/s12889-022-13795-5 PMC9321300

[12] Chaudhary A, Sudzina F, Mikkelsen BE. Promoting healthy eating among Young People-A Review of the evidence of the impact of School-based interventions. Nutrients. 2020;12(9):2894. 10.3390/nu12092894. 32971883 10.3390/nu12092894 PMC7551272

[13] Olarte DA, Koch PA, Wolf RL, Contento IR. Teachers’ resources to support school lunch: Professional Development is warranted. Nutrients. 2022;14(21):4596. 10.3390/nu14214596. 36364866 10.3390/nu14214596 PMC9655880

[14] Story M, Nanney MS, Schwartz MB. Schools and obesity Prevention: creating School environments and policies to promote healthy eating and physical activity. Milbank Q. 2009;87(1):71–100. 10.1111/j.1468-0009.2009.00548.x. 19298416 10.1111/j.1468-0009.2009.00548.x PMC2879179

[15] Center for disease control and prevention (CDC). School Nutrition and the Social and Emotional Climate and Learning | Healthy Schools | CDC. September 21. 2021. Accessed August 16, 2023. https://www.cdc.gov/healthyschools/nutrition/school_nutrition_sec.htm

[16] Dudley DA, Cotton WG, Peralta LR. Teaching approaches and strategies that promote healthy eating in primary school children: a systematic review and meta-analysis. Int J Behav Nutr Phys Activity. 2015;12(1):28. 10.1186/s12966-015-0182-8. 10.1186/s12966-015-0182-8 PMC4416340 25889098

[17] Lee A. Health-promoting schools: evidence for a holistic approach to promoting health and improving health literacy. Appl Health Econ Health Policy. 2009;7(1):11–7. 10.2165/00148365-200907010-00002. 19558191 10.2165/00148365-200907010-00002

[18] Thakur S, Mathur P. Nutrition knowledge and its relation with dietary behaviour in children and adolescents: a systematic review. Int J Adolesc Med Health. 2022;34(6):381–92. 10.1515/ijamh-2020-0192. 33594848 10.1515/ijamh-2020-0192

[19] Rathi N, Riddell L, Worsley A. What influences urban Indian secondary school students’ food consumption? – a qualitative study. Appetite. 2016;105:790–7. 10.1016/j.appet.2016.07.018. 27423818 10.1016/j.appet.2016.07.018

[20] Parker EA, Feinberg TM, Lane HG, et al. Diet quality of elementary and middle school teachers is associated with healthier nutrition-related classroom practices. Prev Med Rep. 2020;18:101087. 10.1016/j.pmedr.2020.101087. 32309116 10.1016/j.pmedr.2020.101087 PMC7155219

[21] Okeyo AP, Seekoe E, de Villiers A, Faber M, Nel JH, Steyn NP. The Food and Nutrition Environment at Secondary Schools in the Eastern Cape, South Africa as reported by Learners. Int J Environ Res Public Health. 2020;17(11):4038. 10.3390/ijerph17114038. 32517072 10.3390/ijerph17114038 PMC7312062

[22] O’Halloran S, Eksteen G, Gebremariam M, Alston L. Measurement methods used to assess the School Food Environment: a systematic review. Int J Environ Res Public Health. 2020;17(5):1623. 10.3390/ijerph17051623. 32138232 10.3390/ijerph17051623 PMC7084932

[23] Moitra P, Madan J, Verma P. Impact of a behaviourally focused nutrition education intervention on attitudes and practices related to eating habits and activity levels in Indian adolescents. Public Health Nutr. 2021;24(9):2715–26. 10.1017/S1368980021000203. 33468283 10.1017/S1368980021000203 PMC10195501

[24] Amahmid O, El Guamri Y, Rakibi Y, et al. Nutrition education in school curriculum: impact on adolescents’ attitudes and dietary behaviours. Int J Health Promotion Educ. 2020;58(5):242–58. 10.1080/14635240.2019.1685399.

[25] Rathi N, Riddell L, Worsley A. Do you think adolescents’ food intake is satisfactory? – views of Indian parents and teachers. Appetite. 2020;153:104740. 10.1016/j.appet.2020.104740. 32428536 10.1016/j.appet.2020.104740

[26] Ministry of Education. Education Statistics Annual Abstract 2019–2020). Ministry Of Education. 2020. Accessed May 10, 2023. http://ecde.aau.edu.et/jspui/handle/123456789/275

[27] Destaw Z, Wencheko E, Kidane S, et al. School feeding contributed valuable dietary energy and nutrients despite suboptimal supply to school-age children and adolescents at primary schools in Addis Ababa, Ethiopia. Nutrition. 2022;102:111693. 10.1016/j.nut.2022.111693. 35816814 10.1016/j.nut.2022.111693

[28] Minten B, Dereje M, Bachewe FN, Tamru S. Evolving Food Systems in Ethiopia: past, Present and Future. 0 ed. International Food Policy Research Institute; 2018. 10.2499/1037800744.

[29] Berhe K, Kidanemariam A, Gebremariam G, Gebremariam A. Prevalence and Associated Factors of Adolescent Undernutrition in Ethiopia: a systematic review and Meta-analysis. BMC Nutr. 2019;5(1):49. 10.1186/s40795-019-0309-4. 32153962 10.1186/s40795-019-0309-4 PMC7050743

[30] Gebrie A, Alebel A, Zegeye A, Tesfaye B, Ferede A. Prevalence and associated factors of overweight/ obesity among children and adolescents in Ethiopia: a systematic review and meta-analysis. BMC Obes. 2018;5(1):19. 10.1186/s40608-018-0198-0. 30002860 10.1186/s40608-018-0198-0 PMC6036672

[31] Kedir S, Hassen K, Melaku Y, Jemal M. Determinants of overweight and/or obesity among school adolescents in Butajira Town, Southern Ethiopia. A case-control study. PLoS ONE. 2022;17(6):e0270628. 10.1371/journal.pone.0270628. 35763506 10.1371/journal.pone.0270628 PMC9239474

[32] Zelalem M, Sinamo S, Maru Y. Meeting the health and nutrition needs of adolescents and youth in Ethiopia. *Nutrition Exchange 13*. Published online March 31, 2020:20. Accessed September 21, 2022. https://www.ennonline.net/nex/13/www.ennonline.net/nex/13/ethiopia

[33] Robinson T. Applying the Socio-ecological model to improving Fruit and Vegetable Intake among Low-Income African americans. J Community Health. 2008;33(6):395–406. 10.1007/s10900-008-9109-5. 18594953 10.1007/s10900-008-9109-5

[34] Moges T, Gebremichael B, Shiferaw S, Yirgu R. Is inadequate play area in schools associated with overweight among students in Addis Ababa, Ethiopia? A comparative cross-sectional study. Epidemiol Health. 2018;40:e2018017. 10.4178/epih.e2018017. 29807411 10.4178/epih.e2018017 PMC6060341

[35] Trines S. Education in Ethiopia. WENR. 2018. Accessed April 8, 2023. https://wenr.wes.org/2018/11/education-in-ethiopia

[36] Shaheen M, Pradhan S, Ranajee R. Sampling in Qualitative Research. In:; 2019:25–51. 10.4018/978-1-5225-5366-3.ch002

[37] Vasileiou K, Barnett J, Thorpe S, Young T. Characterising and justifying sample size sufficiency in interview-based studies: systematic analysis of qualitative health research over a 15-year period. BMC Med Res Methodol. 2018;18(1):148. 10.1186/s12874-018-0594-7. 30463515 10.1186/s12874-018-0594-7 PMC6249736

[38] Devine LD, Gallagher AM, Briggs S, Hill AJ. Factors that influence food choices in secondary school canteens: a qualitative study of pupil and staff perspectives. Front Public Health. 2023;11:1227075. 10.3389/fpubh.2023.1227075. 37522007 10.3389/fpubh.2023.1227075 PMC10375012

[39] Hennink MM, Kaiser BN, Weber MB. What influences saturation? Estimating sample sizes in Focus Group Research. Qual Health Res. 2019;29(10):1483–96. 10.1177/1049732318821692. 30628545 10.1177/1049732318821692 PMC6635912

[40] Edwards-Jones A. Qualitative data analysis with NVIVO. J Educ Teaching: Int Res Pedagogy. 2014;40. 10.1080/02607476.2013.866724.

[41] Braun V, Clarke V. Thematic analysis. SAGE Publications Ltd; 2022.

[42] Trübswasser U, Baye K, Holdsworth M, Loeffen M, Feskens EJ, Talsma EF. Assessing factors influencing adolescents’ dietary behaviours in urban Ethiopia using participatory photography. Public Health Nutr. 2021;24(12):3615–23. 10.1017/S1368980020002487. 32792020 10.1017/S1368980020002487 PMC8369459

[43] Correa N, Rajaraman D, Swaminathan S, et al. Perceptions of healthy eating amongst Indian adolescents in India and Canada. Appetite. 2017;116:471–9. 10.1016/j.appet.2017.05.029. 28529114 10.1016/j.appet.2017.05.029

[44] Silva DC, de A, Frazão IdaS, Osório MM, Vasconcelos MGLde. Perception of adolescents on healthy eating. Cien Saude Colet. 2015;20(11):3299–308. 10.1590/1413-812320152011.00972015. 10.1590/1413-812320152011.00972015 26602708

[45] Koca B, Arkan G. The relationship between adolescents’ nutrition literacy and food habits, and affecting factors. Public Health Nutr Published Online July. 2020;29:1–12. 10.1017/S1368980020001494. 10.1017/S1368980020001494 PMC11574834 32723409

[46] Durão S, Patterson J, Kredo T, Davids E. *Assessing the Existing Evidence Base on School Food and Nutrition Policies: A Scoping Review*.; 2021.

[47] Jacob CM, Hardy-Johnson PL, Inskip HM, et al. A systematic review and meta-analysis of school-based interventions with health education to reduce body mass index in adolescents aged 10 to 19 years. Int J Behav Nutr Phys Act. 2021;18(1):1. 10.1186/s12966-020-01065-9. 33397403 10.1186/s12966-020-01065-9 PMC7784329

[48] Mawela A, van den Berg G. Management of school nutrition programmes to improve environmental justice in schools: a South African case study. South Afr J Clin Nutr. 2020;33(2):30–5. 10.1080/16070658.2018.1507208.

[49] national-nutrition-plan-ethiopia.pdf. Accessed December 19. 2023. https://scalingupnutrition.org/sites/default/files/2022-06/national-nutrition-plan-ethiopia.pdf

[50] Choukem SP, Tochie JN, Sibetcheu AT, Nansseu JR, Hamilton-Shield JP. Overweight/obesity and associated cardiovascular risk factors in sub-saharan African children and adolescents: a scoping review. Int J Pediatr Endocrinol. 2020;2020(1):6. 10.1186/s13633-020-0076-7. 32211050 10.1186/s13633-020-0076-7 PMC7092532

[51] Center for disease control and prevention (CDC). Chapter 1: Models and Frameworks | Principles of Community Engagement | ATSDR. 2015. Accessed May 14, 2023. https://www.atsdr.cdc.gov/communityengagement/pce_models.html

[52] Fox EL, Timmer A. Children’s and adolescents’ characteristics and interactions with the food system. Global Food Secur. 2020;27:100419. 10.1016/j.gfs.2020.100419.

[53] Emergency Nutrition Network (ENN). Adolescent Interest Group Meeting. 2017. Published online February 16, 2018. Accessed June 9, 2023. https://www.ennonline.net/www.ennonline.net/adolescentigmeeting2017

[54] Wrobleski MM, Parker EA, Hager E, et al. Friends and family: how African-American adolescents’ perceptions of Dietary beliefs and behaviors of others relate to Diet Quality. J Acad Nutr Diet. 2018;118(12):2302–10. 10.1016/j.jand.2018.07.021. 30337186 10.1016/j.jand.2018.07.021

[55] Chung A, Vieira D, Donley T, et al. Adolescent peer influence on eating behaviors via Social Media: scoping review. J Med Internet Res. 2021;23(6):e19697. 10.2196/19697. 34081018 10.2196/19697 PMC8212626

[56] Azpiazu L, Antonio-Agirre I, Fernández-Zabala A, Escalante N. How does Social Support and Emotional Intelligence Enhance life satisfaction among adolescents? A Mediational Analysis Study. Psychol Res Behav Manag. 2023;16:2341–51. 10.2147/PRBM.S413068. 37396403 10.2147/PRBM.S413068 PMC10314772

[57] Liu KSN, Chen JY, Ng MYC, Yeung MHY, Bedford LE, Lam CLK. How does the family influence adolescent eating habits in terms of knowledge, attitudes and practices? A global systematic review of qualitative studies. Nutrients. 2021;13(11):3717. 10.3390/nu13113717. 34835973 10.3390/nu13113717 PMC8624651

[58] Totobesola M, Delve R, Nkundimana J, d’Amour, et al. A holistic approach to food loss reduction in Africa: food loss analysis, integrated capacity development and policy implications. Food Sect. 2022;14(6):1401–15. 10.1007/s12571-021-01243-y.

[59] Kraak VI, Vandevijvere S, Sacks G, et al. Progress achieved in restricting the marketing of high-fat, sugary and salty food and beverage products to children. Bull World Health Organ. 2016;94(7):540–8. 10.2471/BLT.15.158667. 27429493 10.2471/BLT.15.158667 PMC4933136

[60] World Health Organization [WHO]. Set of recommendations on the marketing of foods and non-alcoholic beverages to children. 2010. Accessed May 28, 2023. https://www.who.int/publications-detail-redirect/9789241500210

[61] Itria A, Borges SS, Rinaldi AEM, Nucci LB, Enes CC. Taxing sugar-sweetened beverages as a policy to reduce overweight and obesity in countries of different income classifications: a systematic review. Public Health Nutr. 2021;24(16):5550. 10.1017/S1368980021002901. 34218837 10.1017/S1368980021002901 PMC10195460

[62] Carlson SJ, Andrews MS, Bickel GW. Measuring Food Insecurity and Hunger in the United States: development of a National Benchmark measure and prevalence estimates. J Nutr. 1999;129(2):S510–6. 10.1093/jn/129.2.510S. 10.1093/jn/129.2.510S 10064320

[63] Mialon M, Crosbie E, Sacks G. Mapping of food industry strategies to influence public health policy, research and practice in South Africa. Int J Public Health. 2020;65(7):1027–36. 10.1007/s00038-020-01407-1. 32728853 10.1007/s00038-020-01407-1

[64] Kaleab B, Kalle H. Accelerating progress in improving diets and Nutrition in Ethiopia. Intl Food Policy Res Inst; 2020.

[65] El Rhazi K, El Kinany K, Garcia-Larsen V. Chapter 5 - Socioeconomic factors for the adherence to the Mediterranean diet in North Africa: The shift from 1990 to 2019. In: Preedy VR, Watson RR, eds. *The Mediterranean Diet (Second Edition)*. Academic Press; 2020:57–65. 10.1016/B978-0-12-818649-7.00005-9

[66] Cyr-Scully A, Howard AG, Sanzone E, et al. Characterizing the urban diet: development of an urbanized diet index. Nutr J. 2022;21:55. 10.1186/s12937-022-00807-8. 36085037 10.1186/s12937-022-00807-8 PMC9463720

[67] Stamoulis KG, Pingali PL, Shetty P, editors. Emerging Challenges for Food and Nutrition Policy in Developing Countries. *eJADE: electronic Journal of Agricultural and Development Economics*. Published online 2004. 10.22004/ag.econ.12000

[68] Scott S, Elamin W, Giles EL, et al. Socio-Ecological influences on adolescent (aged 10–17) Alcohol Use and Unhealthy Eating behaviours: a systematic review and synthesis of qualitative studies. Nutrients. 2019;11(8):1914. 10.3390/nu11081914. 31443229 10.3390/nu11081914 PMC6722644

[69] Vilar-Compte M, Burrola-Méndez S, Lozano-Marrufo A, et al. Urban poverty and nutrition challenges associated with accessibility to a healthy diet: a global systematic literature review. Int J Equity Health. 2021;20:40. 10.1186/s12939-020-01330-0. 33472636 10.1186/s12939-020-01330-0 PMC7816472

[70] Wrottesley SV, Pedro TM, Fall CH, Norris SA. A review of adolescent nutrition in South Africa: transforming adolescent lives through nutrition initiative. South Afr J Clin Nutr. 2020;33(4):94–132. 10.1080/16070658.2019.1607481.

[71] Tsochantaridou A, Sergentanis TN, Grammatikopoulou MG, Merakou K, Vassilakou T, Kornarou E. Food Advertisement and Dietary choices in adolescents: an overview of recent studies. Child (Basel). 2023;10(3):442. 10.3390/children10030442. 10.3390/children10030442 PMC10047133 36980000

[72] Hawkes C. Uneven dietary development: linking the policies and processes of globalization with the nutrition transition, obesity and diet-related chronic diseases. Global Health. 2006;2:4. 10.1186/1744-8603-2-4. 16569239 10.1186/1744-8603-2-4 PMC1440852

