# Supplementary material for: Teachers’ perception of their students’ dietary habits in Addis Ababa, Ethiopia: a qualitative study
Source: BMC Nutr. 2024 Oct 22;10:141. doi: 10.1186/s40795-024-00946-7 (PMC11494765; doi:10.1186/s40795-024-00946-7)
Supplement: Supplementary file 1 — Supplementary Material 1 [file 40795_2024_946_MOESM1_ESM.docx]

**Focus group discussion guide.**

1. What types of foods and beverages are consumed by the students most of the time?
2. What do you think about the food they eat and beverages they drink?
3. Are there barriers to students consuming a healthy diet? If so, what are these barriers?
4. What do you think influences their food choices?
5. Do you think dietary practices among adolescents have changed compared to your time?
6. What do you think should be done to promote healthy dietary practices among students?

-what kind of interventions are important for students to adopt a healthy diet?

1. Do you have any final comments?

**Observation guide**

1. What foods and beverages are available in the school environment?

-In the nearby cafeteria, shops and food vendors
